# Chloride Influx of Anion Exchanger 2 Was Modulated by Calcium-Dependent Spinophilin in Submandibular Glands

**DOI:** 10.3389/fphys.2018.00889

**Published:** 2018-07-19

**Authors:** Dongun Lee, Sang A. Lee, Dong M. Shin, Jeong H. Hong

**Affiliations:** ^1^Department of Physiology, College of Medicine, Gachon University, Incheon, South Korea; ^2^Department of Oral Biology, College of Dentistry, BK21 PLUS Project, Yonsei University, Seoul, South Korea

**Keywords:** spinophilin, anion exchanger 2, calcium, fluid secretion, salivary glands

## Abstract

Secretory glands including salivary glands by many hormonal inputs produce and secrete biological fluids determined by variety of ion transporters. Spinophilin is a multifunctional scaffolding protein, which involved in receptor signaling and regulation of anion exchangers AE2 activity. We found that spinophilin expressed in salivary glands. The role of salivary spinophilin on the modulation of chloride/bicarbonate exchange remains unknown. The spinophilin enhanced AE2 activity and associated with a STE20/SPS1-related kinase and showed an additive effect on the modulation of the activity of AE2. The cholinergic stimulation and subsequent intracellular Ca^2+^ increase was required for the interaction with AE2 and spinophilin and abrogated the enhanced effect of spinophilin on Cl^−^ transporting activity. Ductal chloride/bicarbonate exchange activity was increased in pretreatment with carbachol. The CaMKII inhibitor KN-93 suppressed the chloride/bicarbonate exchange activity of ducts, suggesting that CaMKII was required for ductal chloride/bicarbonate exchange activity. Additionally, microtubule destabilization by nocodazole attenuated the interaction of AE2 and spinophilin and almost abolished the ductal chloride/bicarbonate exchange activity. The treatment of siRNA-spinophilin on the isolated salivary ducts also reduced the ductal chloride/bicarbonate exchange activity. Therefore, role of salivary spinophilin on AE2 may facilitate the Cl^−^ influx from basolateral in salivary glands in response to cholinergic inputs.

## Introduction

Secretory glands including salivary glands and pancreas by many neuronal, endocrine, and paracrine inputs produce and secrete biological fluids with defined electrolyte composition, determined by many types of ion transporters (Park et al., 2012). The extracellular fluid is rich in Cl^−^ and HCO_3_^−^, which are regulated by a wide variety of channels, including K^+^-Cl^−^ cotransporter KCC, Na^+^-K^+^-Cl^−^ cotransporter NKCC, Na^+^-Cl^−^ cotransporter NCC, Cl^−^/HCO_3_^−^ exchange solute carrier 26 (SLC26) family, Cl^−^/HCO_3_^−^ exchanger AE2, Cl^−^ channel CLC and cystic fibrosis transmembrane conductance regulator (CFTR), and Ca^2+^-activated Cl^−^ channel (CaCC, e.g., TMEM16A). The movement of Cl^−^ and HCO_3_^−^ across the cell plasma membrane has been suggested to be involved in the epithelial cell functions such as pH and volume homeostasis through the basolateral influx and parallel luminal efflux (Lee et al., 2012). Typically, membrane transporters of exocrine glands such as salivary glands and pancreas are localized with polarization. For the movement of Cl^−^, the basolateral NKCC1 and AE2 are involved in Cl^−^ entry, whereas the luminal CFTR and CaCC are associated with Cl^−^ efflux (Lee et al., 2012). For HCO_3_^−^ epithelial secretion, HCO_3_^−^ transport is mediated by the basolateral sodium bicarbonate cotransporter (NBC)-electrogenic isoform (NBCe1), and luminal SLC26 family (e.g., SLC26A6) and electroneutral isoform NBCn1.

Spinophilin (SPL) is a widely distributed scaffold protein involved in the formation of dendritic spines and synaptic activity during neural development (Feng et al., 2000). In addition, SPL plays important functions in the tumor suppression (Ferrer et al., 2011, 2016) and regulation of tumor cell invasion (Cheerathodi et al., 2016). Structural studies show that SPL binds to multiple signaling proteins and receptors including F-actin, several membrane receptors, and protein phosphatase1 (PP1) (Allen et al., 1997; Satoh et al., 1998; Sarrouilhe et al., 2006). As a regulatory subunit of PP1, alteration of SPL-PP1 complex and its functional activities in neurons provide essential significances for synaptic activity (Hsieh-Wilson et al., 2003). The structure of SPL suggests that it is a multifunctional scaffolding protein that regulates both membrane and cytoskeletal functions, including neuronal migration (Sarrouilhe et al., 2006).

The Cl^−^ ion, one of the major intracellular ions, plays a central role in various transport functions, including electrolyte homeostasis and regulation of membrane potential, and acts as a signaling molecule to regulate NBC activity (Kunzelmann et al., 2011; Lee et al., 2012; Shcheynikov et al., 2015). For fluid secretion, the release of neurotransmitter evokes an increase in the intracellular Ca^2+^ concentration, which triggers Cl^−^ efflux. The entry of Cl^−^ by basolateral NKCC and AE and Cl^−^ efflux by luminal CFTR and CaCC draw the tract of Cl^−^ flow.

Plasma membrane AEs regulate cell volume, intracellular pH, base secretion, and intracellular Cl^−^ concentrations (Pena-Munzenmayer et al., 2015). A family of AEs consists of three members, AE1–AE3, expressed in a variety of tissues and Na^+^-independent Cl^−^/HCO_3_^−^ exchanger. Their expression profile has been growing during three decades since AE1 was identified in the erythrocytes (Lux et al., 1989). AE1 is identified in erythrocyte and the renal cortical collecting duct. AE2 is ubiquitously expressed in many epithelia, including kidney, salivary glands and gastrointestinal tract (Vazquez et al., 1995; Roussa et al., 1999). AE3 has been detected in the gut and nervous system and is highly expressed in cardiac tissue (Wang et al., 2014). In salivary glands, AE2 has been localized in parotid, sublingual, and submandibular glands (Zhao et al., 1995; Roussa et al., 1999; Nguyen et al., 2004). The immunostaining of AE2 revealed controversial in salivary ducts, even though its Cl^−^/HCO_3_^−^ activity was present in basolateral membrane of submandibular glands (Zhao et al., 1995). In this study, we found that AE2 localized in the plasma membrane of submandibular ducts. Recently, our previous study revealed the regulatory role of SPL on the AE2, wherein SPL enhances the chloride bicarbonate exchanger (CBE) activity *in vitro* (Jeong and Hong, 2016). However, the role of SPL in the regulation of basolateral AE2 and Cl^−^/HCO_3_^−^ balance in exocrine glands remains unknown. Thus, the mechanism underlying the modulatory functions of SPL should be elucidated in secretory glands.

In the present study, we report that functional SPL is present in the plasma membrane of submandibular gland (SMG) cells. The cholinergic stimulation by carbachol increases the intracellular Ca^2+^ signal and subsequently enhances the CBE activity of SMG acinar cells. Here we found that the intracellular Ca^2+^-dependent role of SPL on the modulation of AE2. The intracellular Ca^2+^ depletion by BAPTA-AM inhibited the interaction with AE2 and SPL and abolished the enhanced effect of SPL on AE2 CBE activity. In the salivary system, the CBE activity of acini cells was unaltered by cholinergic stimulation, while ductal cells were sensitive to BAPTA and Ca^2+^/calmodulin-dependent protein kinase II (Ca^2+^/CaMKII) inhibition. Microtubule destabilization by nocodazole mediated the dissociation of SPL with AE2 and reduced CBE activity in SMG cells. These results suggested that SPL may act as a regulatory protein to preserve HCO_3_^−^-dependent basolateral Cl^−^ influx by cholinergic agonist stimulation in salivary glands.

## Materials and Methods

### Reagents and Plasmids

FLAG (F3165 for mouse monoclonal, F7425 for rabbit polyclonal) and β-actin (A3854) antibodies were purchased from Sigma (St. Louis, MO, United States). Antibodies against SPL (Merck, Germany, AB5669), hemagglutinin (HA; Novus bio, Littleton, CO, United States, NB600-363 for rabbit polyclonal), HA for mouse monoclonal (Cell signaling, #2367) were obtained. GFP antibodies were purchased from Abcam (mouse monoclonal, ab38689) and Santacruz (rabbit, sc-9996). Myc antibody was purchased from Invitrogen (mouse monoclonal, 46-0603). Fura-2-acetoxymethyl ester (Fura-2-AM, 0102) and 2′, 7′-bis-(carboxyethyl)-5-(and-6)-carboxyfluorescein- (BCECF)-AM (0061) were purchased from TEFlabs (Austin, TX, United States). Pluronic acid (F-127, 20% in dimethyl sulfoxide, P3000MP) and *N*-(Ethoxycarbonylmethyl)-6-methoxyquinolinium bromide (MQAE, E3101) were purchased from Invitrogen (Carlsbad, CA, United States). Phorbol 12-myristate 13-acetate (PMA) (16561-29-8) was purchased from Abcam (Cambridge, MA, United States). All other chemicals used were purchased from Sigma. Two types of SPL were used in pCMV-myc and pCMV6-AC-GFP. SPAK (Ste20-related proline alanine rich kinase) and SPAK dominant negative (DN) form of cDNAs were cloned in p3XFLAG-CMV-7.1. All constructs were a kind gift from Dr. Shmuel Muallem (National Institutes of Health, Bethesda).

### Cell Culture

The Human embryonic kidney cells HEK293T and lung adenocarcinoma cell line A549 cells were obtained from the American Type Culture Collection (Rockville, MD, United States, CRM-CCL-185) maintained in Dulbecco’s Modified Eagle’s Medium (Invitrogen, 11995-065) containing 10% FBS (Invitrogen, 16000-044) with 100 U/mL penicillin-streptomycin (Invitrogen, 15140122) during incubating at 37°C in a humidified incubator of 5% CO_2_ and 95% air. When the cells reached 80% confluence, the culture medium was removed, and cells were washed with Dulbecco’s phosphate-buffered saline, followed by their treatment with trypsin/ethylenediaminetetraacetic acid (EDTA) for 2 min. The dispersed cells were transferred to new culture dishes for western blotting and co-immunoprecipitation (Co-IP) or culture dishes with glass coverslips for imaging.

### Isolation of Mouse Submandibular Glands

All experimental protocols for animals, maintenance and care, were conducted according to Gachon University Animal Care guidelines. All animal procedures and protocols were approved by the Center of Animal Care and Use, Lee Gil Ya Cancer and Diabetes Institute, the Institutional Animal Care and Use Committee at Gachon University (Permission number: LCDI-2017-0014). SMG cells were isolated from 8-week old C57BL/6 wild-type mice as previously described (Ji et al., 2017). Briefly, SMG tissues and cells were suspended in HEPES buffer-based physiological salt solution A (PSA (**Table 1**); 140 mM sodium chloride [NaCl], 10 mM glucose, 5 mM potassium chloride [KCl], 1 mM magnesium chloride [MgCl_2_], 1 mM calcium chloride [CaCl_2_], 10 mM HEPES [pH 7.4], 0.02% soybean-trypsin inhibitor, 0.1% sodium pyruvate, and 0.1% bovine serum albumin [BSA]) and stored on ice until use. HEPES buffer-based solution was represented in **Table 2**. Isolated tissues were minced and incubated in collagenase P solution (2.5 mg/10 mL in PSA; Roche, 11213865001) for 6 min at 37°C with vigorous shaking. Following incubation, the products were washed and resuspended in PSA and stored on ice until use.

**Table 1:** Composition of PSA solution.

Composition	Concentration
Sodium chloride (NaCl)	140 mM
HEPES	10 mM
Glucose	10 mM
Potassium chloride (KCl)	5 mM
Magnesium chloride (MgCl_2_)	1 mM
Calcium chloride (CaCl_2_)	1 mM
Soybean-trypsin inhibitor	0.02%
Sodium pyruvate	0.1%
Bovine serum albumin (BSA)	0.1%
pH 7.4	
310 mOsm	

**Table 2:** Composition of HEPES-based solution.

Composition	Concentration
Sodium chloride (NaCl)	140 mM
HEPES	10 mM
Glucose	10 mM
Potassium chloride (KCl)	5 mM
Magnesium chloride (MgCl_2_)	1 mM
Calcium chloride (CaCl_2_)	1 mM
pH 7.4	
300 mOsm (310 for SMG)	

### Treatment With Small Interfering RNA and DNA Transfection

The small interfering RNA (siRNA) for human and mouse SPL was produced using Double-Promoter pFIV-H1/U6 siRNA Cloning and Expression Vectors (System Biosciences, CA, SI111A-1), as per the instructions mentioned on the kit. Purified plasmids contained human siRNA-SPL (sense, 5′-AAA GCC AAC CAA GTG TTC AGC ACT TAC TC-3′ and anti-sense, 5′-AAA AGA GTA AGT GCT GAA CAC TTG GTT GG-3′) and mouse siRNA-SPL (sense, 5′-AAA GAG GAC GAT GAA GAA GAC GAA GAG GA-3′ and anti-sense, 5′-AAA ATC CTC TTC GTC TTC TTC ATC GTC CT-3′). A549 cells and mouse SMG duct were transfected with 1 μg of siRNA vectors. A549 cells expressing native SPL were used for evaluating the siRNA-SPL transfection efficacy. The vectors were diluted in 250 μL Opti-Eagle’s minimum essential media (Opti-MEM^TM^, Invitrogen, 31985-070) and mixed with Lipofectamine 2000 mixture. The mixture was incubated at room temperature for 25 min and transferred into cell dishes containing culture media. After 4 h, transfected media was replaced with the fresh culture media and cells were used 48 h after transfection. Plasmid DNA transfection by Lipofectamine 2000 was followed by manufacturer’s protocol (Invitrogen, 11668019). Each plasmid DNA was diluted in 250 μL of Opti-MEM and 4 μL Lipofectamine 2000 was incubated for 5 min at room temperature with 250 μL of the same medium. The DNA samples and Lipofectamine 2000 were mixed and added to the cell culture dish containing glass coverslip after 25 min. Following 4-h incubation, the medium was replaced with fresh DMEM containing FBS and the cells were used 48 h after transfection.

### Measurement of Intracellular pH for CBE Activity

Transfected cells and isolated SMG cells were attached onto coverslips and loaded in the chamber with 6 μM BCECF-AM in the presence of 0.05% pluronic acid (F-127) for 15 min at room temperature. After stabilization of the fluorescence, the cells were perfused with solution A for at least 5 min prior to intracellular pH (pH_i_) measurements. pH_i_ was measured by BCECF fluorescence using dual excitation wavelengths of 495 and 440 nm and emission wavelength of 530 nm. Ratios of BCECF were converted to pH unit using *in situ* calibration curves as described in Nehrke (2006), Rochon et al. (2007). The measurement of pH calibration proceeded aspirating the calibration solution (**Table 3**) slowly to cells attached on coverslips and incubating room temperature for 5 min. And then, repeat process at pH values 5.5, 6.0, 6.5, 7.0, 7.5, 8.0, and 8.5. The equation of pH calibration curve (**Supplementary Figure S1**) is pH = pKa + log((*R*_max_ − *R*)/(*R* − *R*_min_)) (pKa of BCECF; 6.97, *R*; ratio value of BCECF, *R*_max_; maximum ratio, *R*_max_; minimum ratio). The cells were incubated with CO_2_-saturated HCO_3_^−^-buffered solution (**Table 4**) for the acidification of the cytosol and then perfused with Cl^−^-free HCO_3_^−^-buffered solution (**Table 5**). Arbitrary BCECF fluorescent unit was converted to pH unit followed by the equation of pH calibration curve. CBE activity was calculated from the slope of increase in pH_i_ during the first 30–45 s in Cl^−^-free HCO_3_^−^-buffered solution and expressed as the percent fold change relative to that of CBE activity of control. Fluorescence images were obtained at an interval of 1 s with a CCD camera (Retiga 6000, Q-Imaging, Canada) linked to an inverted microscope (Olympus, Japan) and analyzed with a MetaFluor system (Molecular Devices, PA). Each image was normalized by subtracting the background fluorescence from the raw background signals.

**Table 3:** pH Calibration solution.

Composition	Concentration
Potassium chloride (KCl)	135 mM
HEPES	20 mM
Dipotassium phosphate (K_2_HPO_4_)	10 mM
Calcium chloride (CaCl_2_)	1.2 mM
Magnesium sulfoxide (MgSO_4_)	1 mM
Nigericin	20 μM
Adjusting pH to 5.5–8.5 (0.5 Interval)	

**Table 4:** Composition of HCO_3_^−^-buffered solution.

Composition	Concentration
Sodium chloride (NaCl)	120 mM
HEPES	2.5 mM
Glucose	10 mM
Potassium chloride (KCl)	5 mM
Magnesium chloride (MgCl_2_)	1 mM
Calcium chloride (CaCl_2_)	1 mM
Sodium bicarbonate (NaHCO_3_)	25 mM
pH 7.8	
300 mOsm (310 for SMG)	

**Table 5:** Composition of Cl^−^-free HCO_3_^−^-buffered solution.

Composition	Concentration
Sodium gluconate	120 mM
HEPES	2.5 mM
Glucose	10 mM
Potassium gluconate	5 mM
Magnesium sulfoxide (MgSO_4_)	1 mM
Calcium gluconate	1 mM
Sodium bicarbonate (NaHCO_3_)	25 mM
pH 7.8	
300 mOsm (310 for SMG)	

### Intracellular Ca^2+^ Concentration Measurement

The intracellular Ca^2+^ concentration ([Ca^2+^]_i_) was determined by Fura-2-AM fluorescence ratios using double excitation wavelengths of 340 and 380 nm (*R*_340/380_) and emission wavelength of 510 nm. Isolated SMG cells were incubated with 10 μM Fura-2-AM in the presence of 0.05% pluronic acid (F-127) for 15 min at room temperature and transferred onto coverslips. Fluorescence images were obtained at an interval of 1 s by a CCD camera (Q-Imaging) linked to an inverted microscope with perfusing HEPES buffer-based solution (**Table 2**) and analyzed with a MetaFluor system (Molecular Devices). Each image was normalized by subtracting the background fluorescence from the raw background signals.

### Measurement of Intracellular Cl^−^ Transporting Activity

Intracellular Cl^−^ was evaluated from *N*-(Ethoxycarbonylmethyl)-6-methoxyquinolinium bromide (MQAE, Thermo, E-3101) fluorescence as previously described (Park et al., 2013; Jeong and Hong, 2016). HEK293T and primary isolated SMG cells seeded on coverslips were treated with 5 mM MQAE for 30 min at room temperature and perfused with NaCl-based solution until signal stabilization. Fluorescence was recorded for at least 5 min to obtain a steady baseline. The perfusion solution was replaced with 0 mM Cl^−^, followed by the addition of HCO_3_^−^ solution containing 126 mM Cl^−^. MQAE fluorescence was recorded at an excitation and emission wavelength of 360 and 510 nm, respectively, with a CCD camera attached to an inverted microscope. MQAE is a quenching dye; therefore, an increase in the MQAE fluorescence unit corresponds to a decrease in the intracellular Cl^−^ concentration, while the decrease in the fluorescence unit reflects the increase in Cl^−^ concentration. Due to the lack of MQAE dye calibration, fluorescence units are unsuitable for the estimation of absolute Cl^−^ secretion. The slope of the increase in MQAE fluorescence during the first 30–45 s indicates Cl^−^ transporting activity in Cl^−^-free HCO_3_^−^-buffered media and is expressed as the percent fold change relative to the Cl^−^ transporting activity of control. Images were analyzed with a MetaFluor system (Molecular Devices).

### Co-immunoprecipitation, Surface Biotinylation, and Western Blotting

Transfected cells were incubated with lysis buffer (Cell signaling, 9803) containing 20 mM Tris, 150 mM NaCl, 2 mM EDTA, 1% Triton X-100, and a protease inhibitor mixture for 5 min at room temperature. The cells were sonicated and centrifuged at 11,000 ×*g* for 15 min at 4°C and protein concentration was determined by Bradford assay (Bio-Rad, 5000001). For the Co-IP, the supernatant was treated with 1 μg/mL indicated antibodies at 4°C for 16 h with gentle shaking, followed by its incubation with 80 μl agarose G protein beads (Santa Cruz, SC-2002) for 4 h. The mixture was centrifuged at 11,000 × *g* for 2 min at 4°C and washed twice with the lysis buffer at 4°C for 10 min. The beads were incubated at 37°C for 15 min for protein detachment. Eluted proteins were performed western blotting. To demonstrate the surface expression of proteins, transfected cells were incubated with 0.5 mg/mL EZ-LINK Sulfo-NHS-LC-biotin (Thermo, 21335) for 30 min on ice, followed by their treatment with 100 mM cold glycine solution for 10 min. Incubated cells were washed with phosphate-buffered saline (PBS) and incubated with the lysis buffer. Cell extracts were centrifuged at 11,000 ×*g* for 15 min at 4°C. The supernatants were overnight incubated with 100 μL Avidin beads (Thermo, 20347) at 4°C, followed by washing of the beads with the lysis buffer. Collected beads were heated at 37°C for 15 min to recover proteins. The warmed protein samples (30 μg) were subjected to separation using sodium dodecyl sulfate polyacrylamide gel electrophoresis (SDS-PAGE) and then transferred onto polyvinylidene difluoride (PVDF, Bio-Rad, 1620177) membranes soaked in methanol. The membrane was blocked with 5% non-fat milk solution in TBS-T [Tris-buffered saline (TBS) and 0.5% Tween-20] for 1 h. The membrane was incubated with indicated antibodies overnight at 4°C and washed thrice with TBS-T. Following washing, membranes were incubated with horseradish peroxidase (HRP)-conjugated anti-mouse and anti-rabbit secondary antibodies and the protein bands visualized using the enhanced luminescent solution (Thermo, 32209).

### Immunofluorescence and Confocal Imaging

Isolated SMG cells and sliced SMG tissues were transferred onto cover glasses and fixed with chilled methanol (-20°C, 10 min for AE2, ZO-1, and SPL) or 4% paraformaldehyde (room temperature (RT), 10 min for NBCe1 and NKCC1). Formaldehyde aqueous solution (16%, Electron Microscopy Sciences, Hatfield, PA, United States, 30525-89-4) was diluted to 4% with PBS at RT. After three times washing for 10 min, fixed cells were overnight incubated with primary antibodies (1: 100 dilution factor) at 4°C, followed by washing thrice with PBS. To detect bound antibodies, cells were treated with secondary antibodies (1: 200 dilution factor), goat immunoglobulin G (IgG)-tagged with rhodamine (Jackson ImmunoResearch, anti-mouse: 115-025-072, anti-rabbit: 111-025-144) or fluorescein isothiocyanate (FITC, Jackson ImmunoResearch, anti-mouse: 115-095-071, anti-rabbit: 111-095-003), for 1 h. Cells were mounted on glass slides using Fluoromount-G^TM^ with 4′,6-diamidino-2-phenylindole (DAPI) (Electron Microscopy Sciences, 17984-24) and analyzed using LSM 700 Zeiss confocal microscope (Carl Zeiss, Germany) with ZEN software (Carl Zeiss).

### Statistical Analyses

Data from indicated number of experiments were expressed as mean ± standard error of the mean (SEM). Comparisons among groups were performed using one-way analysis of variance (ANOVA). A two-sided *P*-value (^∗^*P* < 0.05, ^∗∗^*P* < 0.01, ^∗∗∗^*P* < 0.001) was considered to be statistically significant.

## Results

### The Localization of Anion Exchanger AE2 and Salivary SPL

**FIGURE 1:**
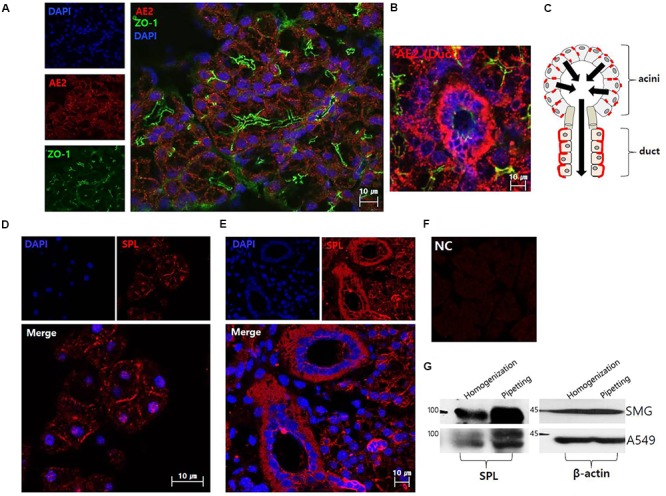
The localization of anion exchanger AE2 and salivary SPL. **(A)** The plasma membrane expression of native AE2 in SMG tissues. Co-staining with ZO-1 (green), AE2 (red), and DAPI (nucleus, blue) in SMG tissues. **(B)** Ductal expression of AE2 (red). **(C)** The figure was illustrated the localization of AE2 in salivary acini and ductal cells. Black arrows represent the secretion flow. Immunostaining of SPL (red) and DAPI (blue) in isolated SMG acini **(D)** and SMG tissues **(E)**. **(F)** Negative control (NC) of immunofluorescence image without primary SPL antibody of SMG tissue. **(G)** Protein expression of SPL in isolated SMG cells and A549 cell line with different protocol of cell lysis. The A549 cells were used as a positive control for SPL expression. The β-actin blot was used as a loading control.

**Figure 1A** shows the plasma membrane expression of native AE2 in SMG tissues. Co-staining with tight junction marker ZO-1 revealed that AE2 localized in basolateral membrane. SMG duct also expressed AE2 in basolateral membrane (**Figure 1B**). The **Figure 1C** illustrated the localization of AE2 in salivary acini and ductal cells. Isolated SMG acini and SMG tissues revealed that for the first native SPL was expressed in lateral membrane and strongly expressed in the SMG duct (**Figures 1D,E**). Immunostaining images were verified with negative control image (**Figure 1F**). In addition, expression of SPL protein in SMG was verified with western blotting. To get the protein sample effectively, cells were lysed with different isolation protocol (**Figure 1G**). Lysis with pipetting obtained more sample fraction. Lysate of A549 cells was used a positive control of SPL expression.

### Molecular Interaction and SPL-Mediated CBE Activity of AE2 and SPAK

**FIGURE 2:**
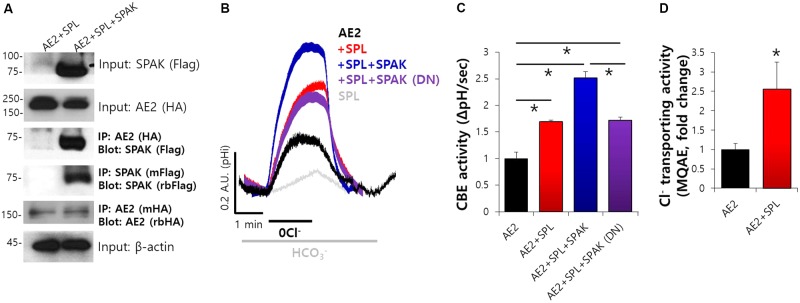
Molecular interaction and SPL-mediated CBE activity of AE2 and SPAK. **(A)** The interaction between AE2 and SPAK through SPL in HEK293T cells with the indicated plasmids. The brackets represent the detected antibodies. β-actin and input blots were used as loading control. **(B)** Changes in pH_i_ of AE2 in HEK293T cells transfected with the indicated plasmids. **(C)** Bars indicate the means ± SEM of the number of experiments. The effect of the indicated proteins on AE2 was normalized to activity of AE2 in the control (black). (^∗^*P* < 0.05, *n* = 6). SPAK (DN): SPAK dominant negative. **(D)** Cl^−^ transporting activity with MQAE technique in AE2 with or without SPL in HEK293T cells. Bars indicate the means ± SEM of the number of experiments (^∗^*P* < 0.05, *n* = 4).

Our previous study showed that SPL interacts with AE2 and enhances its activity (Jeong and Hong, 2016). We determined the specific regulatory proteins involved in SPL machinery. The analysis of the amino acid sequence revealed that SPL contains a Ste20p-related proline alanine-rich kinase (SPAK)-docking motif (577-580: R/K-F-X-V/I) (Jeong and Hong, 2016). As shown in **Figure 2A**, Co-IP data revealed that SPL interacts with SPAK. Previous report has been verified that AE2 alone did not bind to SPAK (Jeong and Hong, 2016). To elucidate the regulatory role of SPL and its binding partners, CBE activity of AE2 was evaluated by measuring the change in the pH_i_ induced by Cl^−^ removal from HCO_3_^−^-buffered solution. The removal of Cl^−^ from the medium induced intracellular alkalization in AE2-expressing cells. In the presence of SPL and SPAK, CBE activity of AE2 dramatically increased and the dominant negative form of SPAK abrogated this effect (**Figures 2B,C**). Additionally, we measured the regulatory role of SPL on AE2 activity by evaluating Cl^−^ transport using Cl^−^-sensitive dye MQAE. The movement of Cl^−^ by AE2 resulted in the de-quenching of MQAE fluorescence. Consistent with these findings, we observed an increase in Cl^−^ transporting activity of AE2 in the presence of SPL (**Figure 2D**). We also checked the role of SPL on the basolateral Cl^−^ and HCO_3_^−^ transporters, expressed in submandibular glands, NBCe1-B and NKCC1 (**Supplementary Figure S2A**). Neither NBCe1-B nor NKCC1 did bind to SPL (**Supplementary Figures S2B,C**). Activities of NBCe1-B and NKCC1 were also independent on SPL (**Supplementary Figures S2D,E**).

### Carbachol-Induced Ca^2+^ Increase Mediates Enhanced Ductal CBE Activity

**FIGURE 3:**
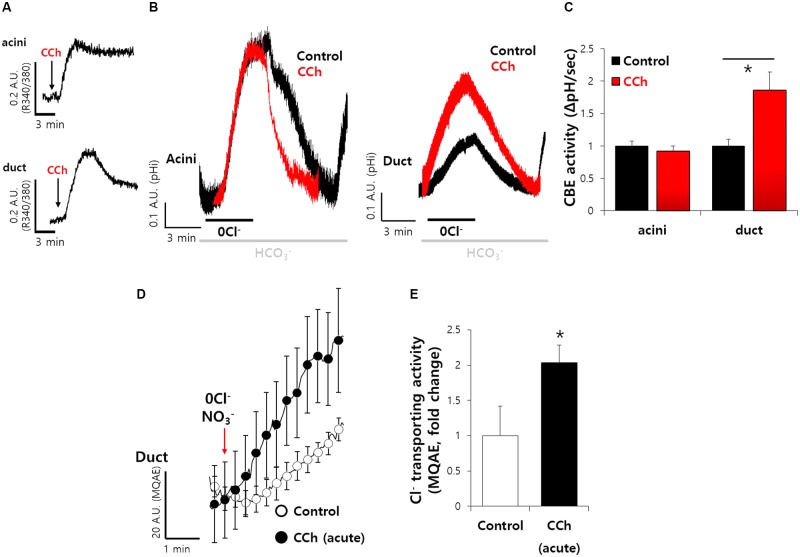
Carbachol-induced Ca^2+^ increase mediates enhanced ductal CBE activity. **(A)** The cholinergic receptor agonist carbamyl choline chloride (carbachol, 1 μM)-evoked intracellular Ca^2+^ signals in SMG acini and ductal cells. Arrows represent the applied time of carbachol. **(B)** The effect of acute carbachol on CBE activities of SMG acini and ductal cells. **(C)** Bars indicate the means ± SEM of the number of experiments (^∗^*P* < 0.05, *n* = 5). **(D)** Changes Cl^−^ transporting activity by carbachol (1 μM) with MQAE quenching technique in isolated SMG ductal cells. **(E)** Bars indicate the means ± SEM of the number of experiments (^∗^*P* < 0.05, *n* = 3).

Our previous study showed that SPL interacts with AE2 and enhances its activity (Jeong and Hong, 2016). We evaluated the mechanism underlying SPL-mediated modulation of CBE activities. SPL is phosphorylated by Ca^2+^/CaMKII (Grossman et al., 2004), which is abundant in neurons and acts as a cardinal protein in neurotransmission and synaptic plasticity (Lisman et al., 2002). We verified the effect of SPL on transporters following its activation via Ca^2+^-dependent CaMKII. The activation of CaMKII is induced by the stimulation of cholinergic receptors, which induce gastric acid secretion in mucosal glands (Fahrmann et al., 2002a,b). To verify the physiological effect of the activation of sympathetic nervous system, which involves cholinergic receptors in salivary glands, SMG cells were stimulated with carbamylcholine chloride (carbachol) to increase the intracellular Ca^2+^. Stimulation of muscarinic receptor by carbachol enhances HCO_3_^−^ secretion (Takeuchi et al., 2015). The acute administration of carbachol induced a transient increase in the intracellular Ca^2+^ level (**Figure 3A**). For CBE activity in the presence of acute carbachol stimulation, no change in slope was observed in acinar cells, whereas twofold enhanced CBE activity was observed in ductal cells (**Figures 3B,C**), suggesting that the increased Ca^2+^ by carbachol mediates enhanced ductal CBE activity to secrete HCO_3_^−^. To examine this phenomenon *in vivo*, the isolated ducts were perfused with HEPES buffer-based solution in which NO_3_^−^ is a substituent of Cl^−^, resulting in an increase in MQAE fluorescence (Yang et al., 2009; Park et al., 2013). Increased MQAE fluorescence reflected the enhanced Cl^−^ secretion. Stimulation with carbachol markedly enhanced MQAE fluorescence in isolated ducts (**Figures 3D,E**).

### SPL-Mediated Increase in AE2 Activity Was Dependent on the Intracellular Ca^2+^

**FIGURE 4:**
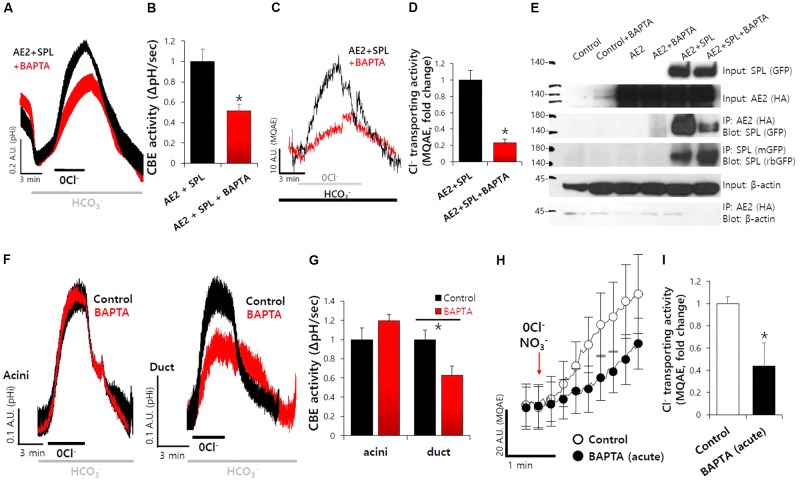
SPL-mediated increase in AE2 activity was dependent on the intracellular Ca^2+^. **(A)** The effect of free Ca^2+^ chelator BAPTA-AM (BAPTA, 10 μM, 1 h) on SPL-mediated CBE activity and Cl^−^ transporting activity of AE2, respectively, in HEK293T cells. **(B)** Bars indicate the means ± SEM of the number of experiments. The effect of BAPTA on AE2 with SPL was normalized to the activity of the control (black). (^∗^*P* < 0.05, *n* = 5). **(C)** Cl^−^ transporting activity with MQAE technique in AE2 with or without SPL in HEK293T cells. **(D)** Bars indicate the means ± SEM of the number of experiments (^∗^*P* < 0.05, *n* = 4). **(E)** The interaction between AE2 and SPL with and without BAPTA-AM in HEK293T cells. The brackets represent the detected antibodies. **(F)** The effect of BAPTA-AM (10 μM) for 1 h on CBE activities of isolated SMG acini and ductal cells. **(G)** Bars indicate the means ± SEM of the number of experiments (^∗^*P* < 0.05, *n* = 4). **(H)** Changes Cl^−^ transporting activity by BAPTA-AM (10 μM) with MQAE quenching technique in isolated SMG ductal cells. **(I)** Bars indicate the means ± SEM of the number of experiments (^∗^*P* < 0.05, *n* = 3).

To verify the effect of SPL on transporters following its activation via Ca^2+^-dependent CaMKII, cells were treated with BAPTA-AM for Ca^2+^ depletion. We found that SPL-mediated increase in CBE activity and Cl^−^ transporting activity of AE2 were abolished in the absence of Ca^2+^, respectively (**Figures 4A–D**). We evaluated the protein interaction of AE2 with SPL with or without BAPTA-AM treatment. The intracellular Ca^2+^ depletion mediated by BAPTA-AM suppressed the interaction between AE2 and SPL (**Figure 4E**). To verify the role of Ca^2+^ depletion on CBE activity, isolated SMG cells were measured CBE activity in the presence of BAPTA-AM. Ductal CBE activity was inhibited by about 50% by the BAPTA-AM treatment whereas no effect of BAPTA-AM on acini (**Figures 4F,G**). To evaluate the Ca^2+^-dependent Cl^−^ transporting activity *in vivo*, the isolated ducts were perfused with HEPES buffer-based solution in which NO_3_^−^. Stimulation with BAPTA-AM inhibited MQAE fluorescence in isolated SMG ducts (**Figures 4H,I**). BAPTA-AM also inhibited carbachol-induced Ca^2+^ signals in SMG acinar and ductal cells (**Supplementary Figures S3A,B**). Carbachol-induced ductal CBE activity was dramatically inhibited by the BAPTA-AM (**Supplementary Figures S3C,D**). These results suggested that ductal CBE activity was dependent on intracellular Ca^2+^ level.

### Ductal CBE Activity Was Mediated by CaMKII

**FIGURE 5:**
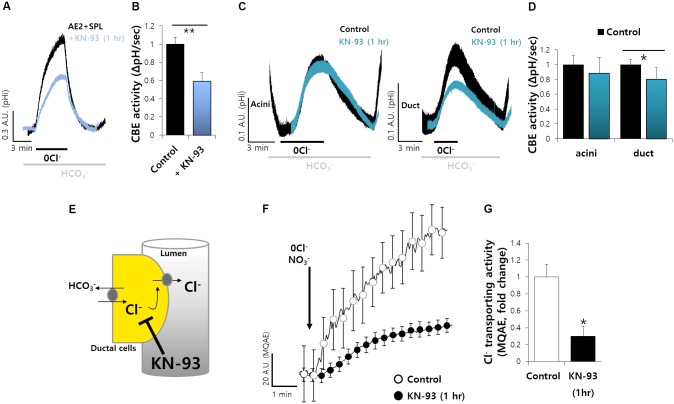
Ductal CBE activity was mediated by CaMKII. **(A)** The effect of KN-93 (20 μM) on CBE activities of AE2 and SPL co-expressed cells for 1 h in HEK293T cells. **(B)** Bars indicate the means ± SEM of the number of experiments (^∗∗^*P* < 0.01, *n* = 4). **(C)** Changes in pH_i_ of isolated SMG acini and ductal cells with and without KN-93. **(D)** Bars indicate the means ± SEM of the number of experiments (^∗^*P* < 0.05, *n* = 4). **(E)** Figures represent the inhibition model of Cl^−^ flux by KN-93. **(F)** Changes Cl^−^ transporting activity by KN-93 (20 μM) with MQAE technique in isolated SMG ductal cells. **(G)** Bars indicate the means ± SEM of the number of experiments (^∗^*P* < 0.05, *n* = 4).

Spinophilin is phosphorylated by Ca^2+^/CaMKII (Grossman et al., 2004). To verify the role of CaMKII-dependent SPL, cells were co-transfected with AE2 and SPL. The cells were measured CBE activity with and without CaMKII inhibitor KN-93. The CBE activity was inhibited by about 50% in the presence of KN-93 (**Figures 5A,B**). Additionally, in salivary system, we pretreated SMG cells with KN-93 and found that the activity of ductal CBE, not acinar CBE, was inhibited by about 25% (**Figures 5C,D**). Thus, CaMKII is required for CBE activity in the duct. Basolateral Cl^−^ influx mediates Cl^−^ secretion by CFTR to draw the tract of Cl^−^ flow. To verify the *in vivo* effect of CaMKII on CBE activity, we speculated that inhibition of Cl^−^ influx by KN-93 may attenuate the Cl^−^ efflux by Cl^−^ efflux channel such as CFTR (**Figure 5E**). To test this hypothesis *in vivo*, the isolated ducts were applied with HEPES buffer-based solution in which NO_3_^−^. Stimulation with KN-93 markedly inhibited MQAE fluorescence, which means inhibited Cl^−^ secretion, in isolated ducts, similar with AE2+SPL-overexpressed cells *in vitro*, suggesting that ductal CBE activity is dependent on CaMKII (**Figures 5F,G**).

### Microtubule Stabilization Maintain the Ductal CBE Activity

**FIGURE 6:**
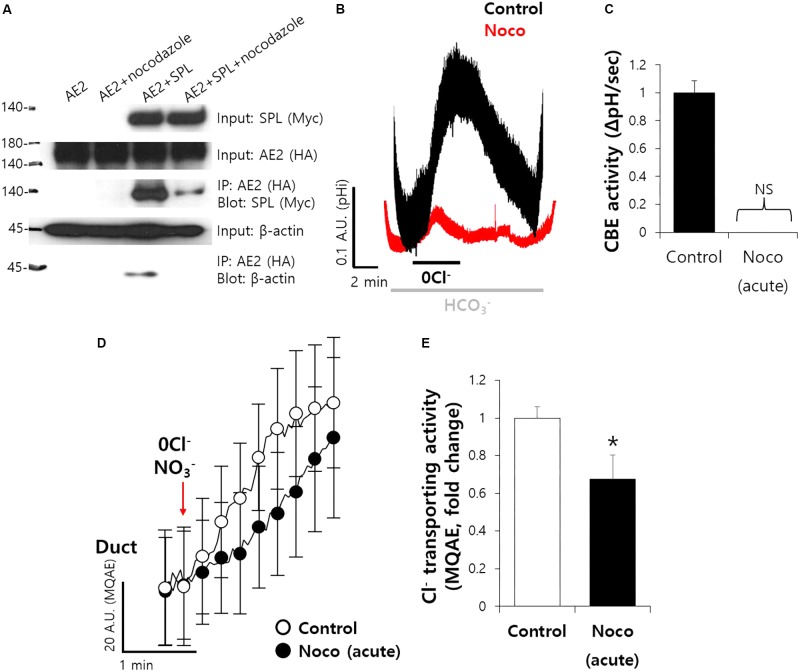
Microtubule stabilization maintain the ductal CBE activity. **(A)** The interaction between AE2 and SPL with and without nocodazole (10 μM, 1 h) in HEK293T cells. The brackets represent the detected antibodies. β-actin was used as a loading control. **(B)** Changes in pH_i_ of isolated SMG ductal cells with and without nocodazole (Noco). **(C)** Bars indicate the means ± SEM of the number of experiments (*n* = 4). NS, not stipulated. **(D)** Changes Cl^−^ transporting activity by nocodazole (10 μM) with MQAE quenching technique in isolated SMG ductal cells. **(E)** Bars indicate the means ± SEM of the number of experiments (^∗^*P* < 0.05, *n* = 3).

To explore the molecular mechanism of ductal CBE enhancer SPL, we treated microtubule destabilizer. SPL involves in microtubule stabilization (Kalil et al., 2000; Bielas et al., 2007). Nocodazole, an inhibitor of integration of membrane microtubules (Alves-Silva et al., 2017), reduced the protein interaction between SPL and AE2 (**Figure 6A**). To verify the effect of microtubule destabilization in SMG, isolated SMG ductal cells were treated with nocodazole. Ductal CBE activity was almost abolished in the treatment of nocodazole (**Figures 6B,C**). For the confirmation of Cl^−^ transporting activity in isolated SMG ductal cells, MQAE quenching technique was applied. The Cl^−^ secretion was also inhibited by nocodazole (**Figures 6D,E**).

### SiRNA-SPL Mediated the Reduced Ductal CBE Activity

**FIGURE 7:**
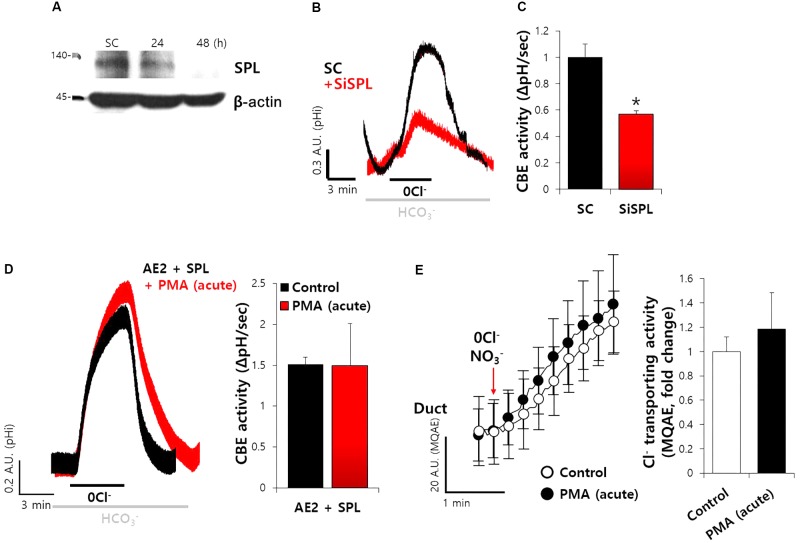
SiRNA-SPL mediated the reduced ductal CBE activity. **(A)** Knockdown efficiency of SPL in A549 cells. The protein expression of SPL and β-actin with and without siRNA-SPL. **(B)** The traces of siRNA-SPL (siSPL) after treatment for 48 h were evaluated by measuring the change in the pH_i_ of isolated SMG ductal cells. The slope of pH_i_ measured CBE activity in the absence of Cl^−^ at the beginning of time course (30–45 s) and the height to reach the point of maximum pH_i_ from the minimum point. **(C)** Bars represent the mean ± SEM of the number of experiments (^∗^*P* < 0.05, *n* = 4). SC, scrambled control of siRNA-SPL. **(D)** The traces and slope of pH_i_ measured CBE activity with and without PMA (50 ng/mL) in AE2+SPL-overexpressed HEK293T cells (*n* = 3). **(E)** Changes Cl^−^ transporting activity by PMA (50 ng/mL) with MQAE quenching technique in isolated SMG ductal cells. Bars indicate the means ± SEM of the number of experiments (*n* = 3).

To explore the effect of SPL on CBE activity in SMG additionally, we developed the siRNA cloning and expression vector system of human and mouse SPL. Secretory acinar cells, including salivary acini, are highly polarized and lose their polarity within 12 h in experimental cultures. To avoid the experimental limitation, the efficiency of siRNA vector system was evaluated in A549 cells expressing native SPL, owing to the experimental limitation associated with the cultured salivary glands (**Figure 7A**). Thus, siRNA-SPL can be applied in cultured duct system. Given the importance of SPL-mediated modulation of CBE activity in siRNA-treated ductal cells, we hypothesized the involvement of SPL and CBE activities in salivary gland fluid and HCO_3_^−^ secretion. The knockdown of SPL for 48 h resulted in about 60% decrease in the CBE activity in isolated ducts (**Figures 7B,C**). To confirm the role of AE2 on CBE activity in SMG, isolated SMG cells were treated with protein kinase C agonist phorbol 12-myristate 13-acetate (PMA), known as a luminal CBE inhibitor (Hassan et al., 2007). Neither CBE activity of AE2+SPL-overexpressed cells nor the Cl^−^ secretion by MQAE quenching technique in SMG duct was not inhibited by the PMA (**Figures 7D,E**). Although the role of SPL on SMG acinar cells was not addressed because of experimental limitation, these results suggest that SPL involves in the regulation of ductal CBE.

## Discussion

The bulk of fluid secretion occurs in secretory glands through a coordinated function of a variety of basolateral and luminal transporters. AE2 acts as key exchanger of Cl^−^ influx and HCO_3_^−^ efflux in the basolateral membrane. Here we addressed the new role of the scaffolding protein SPL in the regulation of AE2 CBE activity and CBE activity of salivary ducts. We have previously shown that AE2 supplies HCO_3_^−^-dependent Cl^−^ for fluid secretion in the salivary duct (Hong et al., 2015). After hormonal inputs such as the release of neurotransmitters, the salivary gland cells, including ductal cells, increase the intracellular Ca^2+^, resulting in the fluid and enzyme secretion. The initial secretion contains Cl^−^-rich fluid. The main function of salivary SPL in the ductal fluid secretion is to enhance AE2 activity to induce Cl^−^ influx. The Cl^−^ transporting activity in the MQAE quenching technique by this transporter also revealed the enhanced effect in the presence of SPL.

In the current study, we addressed that the regulation and interaction of AE2 with SPL is sensitive to intracellular Ca^2+^ concentration. Nguyen et al. (2004) addressed that AE2 mediated the main portion of the CBE across the basolateral membrane of mouse parotid and sublingual glands and was dependent on muscarinic receptor-mediated Ca^2+^ increase. Although our results do not allow us to explain what the portion of other AEs contributes to this mechanism, the SPL enhances the Cl^−^ influx by basolateral AE2 in the SMG duct during the agonist-stimulated secretory stage to facilitate the secretion of Cl^−^ and the CBE activity.

It is not surprising that the secretory flow of Cl^−^ is from basolateral to luminal direction by CaCC (e.g., TMEM16A) for acinar cells and CFTR for ducts; however, the present study strongly suggests that basolateral Cl^−^ uptake was modulated by the association of SPL-mediated CBEs and may facilitate the Cl^−^ secretion in response to agonist stimulation (**Figure 6**). In secretory ducts, luminal solute carrier 26 family A6 (SLC26A6) is also involved in Cl^−^ influx (Ko et al., 2004). Not only basolateral AE2 but also luminal SLC26A6 might be modulated by SPL. The identification of role of SPL on SLC26A6 raises the possibility in the regulation of epithelial transporters will be of interest.

The various roles of the SPL complex suggest that the multifunctional scaffolding protein regulates glutamate receptor function, neuronal migration, seven-transmembrane domain receptor signaling, and Rac G protein guanine nucleotide exchange factor (GEF) signaling (Sarrouilhe et al., 2006). In this report, we provide the extended role of SPL in the regulation of CBEs. Secretory ducts possess variety of Cl^−^ transporters such as SLC26A and CFTR. The regulatory role of SPL between basolateral transporters and luminal transporters should be elucidated in ductal cells to evaluate the fidelity of electrolyte movements. Agonist stimulation or neuronal inputs induce the increase of Ca^2+^, and subsequently, the enhanced AE2 activity by SPL may mediate additional intracellular Cl^−^ intake, although Cl^−^ influx is mediated by various transporters including NKCC1. The enhanced intracellular Cl^−^ concentration may affect the activity of SPAK. Subsequently, the SPAK activation causes the change of CFTR characteristics, from Cl^−^ efflux-preferring channel to HCO_3_^−^ efflux-preferring channel (Park et al., 2010). Thus, intracellular Cl^−^ level will be preserved until Cl^−^ efflux channels are activated. The presence of salivary SPL and its association with AE2 provides the basolateral Cl^−^ intake and may maintain Cl^−^ flow for programmed ductal Cl^−^ secretion during agonist stimulation.

## Author Contributions

JH, SL, and DL contributed to conception and design, acquisition of data, or analysis and interpretation of data. SL and DL made drafting the article or revising it critically for important intellectual content. JH and DS contributed final approval of the version to be published agreement to be accountable for all aspects of the work in ensuring that questions related to the accuracy or integrity of any part of the work are appropriately investigated and resolved.

## Conflict of Interest Statement

The authors declare that the research was conducted in the absence of any commercial or financial relationships that could be construed as a potential conflict of interest.

## Supplementary Material

FIGURE S1 The pH calibration curve for **(A)** HEK293T, **(B)** A549, and primary isolated SMG **(C)** acini and **(D)** ductal cells at pH 5.5, 6.0, 6.5, 7.0, 7.5, 8.0, and 8.5.

FIGURE S2 Expression of NBCe1 (red) and NKCC1 (red) in isolated SMG cells. The polyclonal NBCe1 antibody can recognize shared epitope on the NBCe1-A, -B, and -C isoform. **(B)** The interaction between NBCe1-B and SPL. Input blots were for loading control. **(C)** The interaction between NKCC1 and SPL. Input blots were for loading control. **(D,E)** The slope of pH_i_ measured CBE activity in the absence of Cl^−^ with indicated plasmids. Bars represent the mean ± SEM of the number of experiments (*n* = 3).

FIGURE S3 The cholinergic receptor agonist carbachol (1 μM)-evoked intracellular Ca^2+^ signals with and without BAPTA-AM (BAPTA, 10 μM) in SMG acini **(A)** and ductal **(B)** cells. Arrows represent the applied time of agents. **(C)** The effect of acute carbachol with and without BAPTA-AM (10 μM) on CBE activities of SMG ductal cells. **(D)** Bars indicate the means ± SEM of the number of experiments (^∗^*P* < 0.05, *n* = 3).
