# Supplementary material for: Chloride Influx of Anion Exchanger 2 Was Modulated by Calcium-Dependent Spinophilin in Submandibular Glands
Source: Front Physiol. 2018 Jul 19;9:889. doi: 10.3389/fphys.2018.00889 (PMC6060233; doi:10.3389/fphys.2018.00889)

Supplementary Figure S1. Lee and Lee et al.

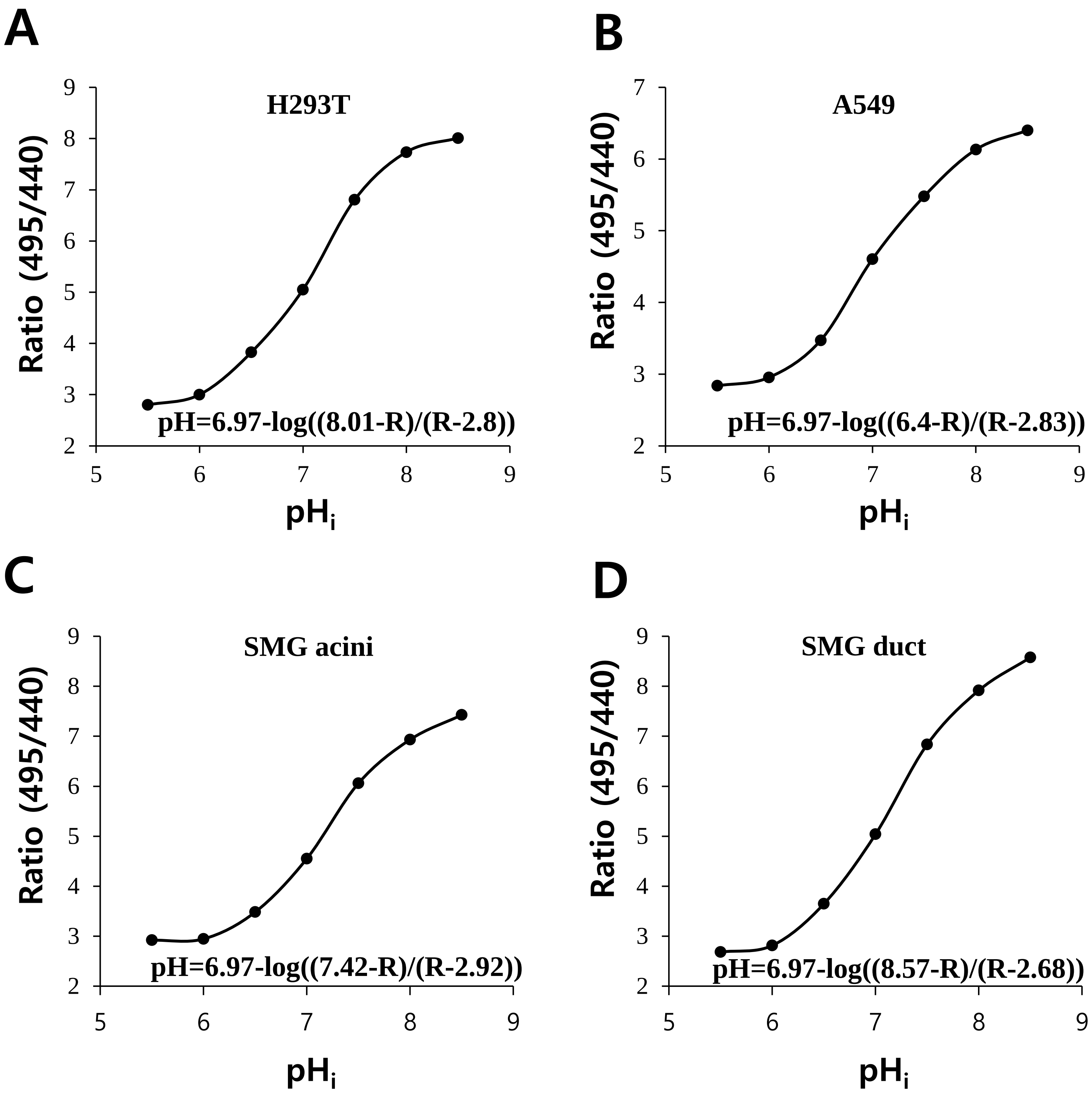

Supplementary Figure S2. Lee and Lee et al.

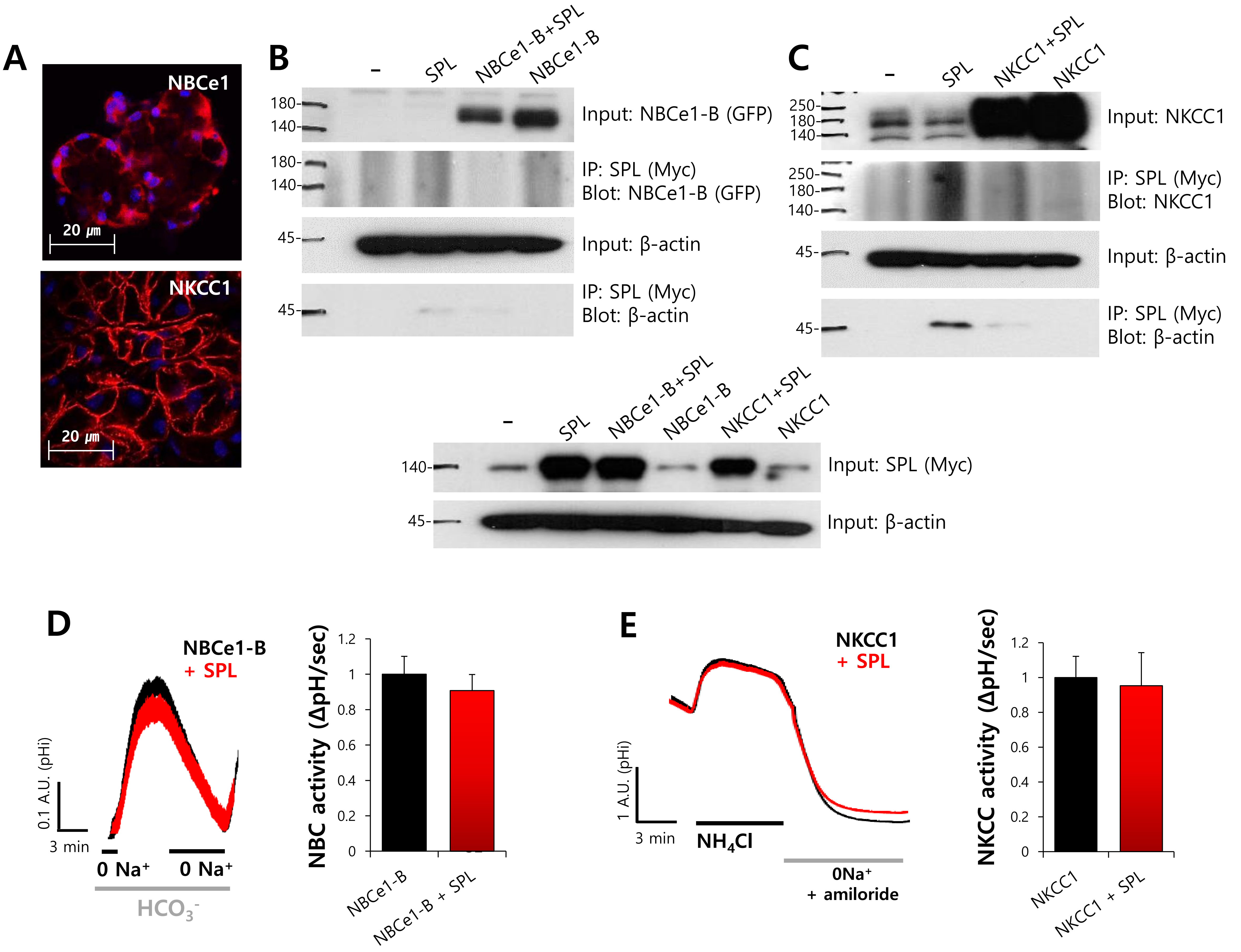

Supplementary Figure S3. Lee and Lee et al.

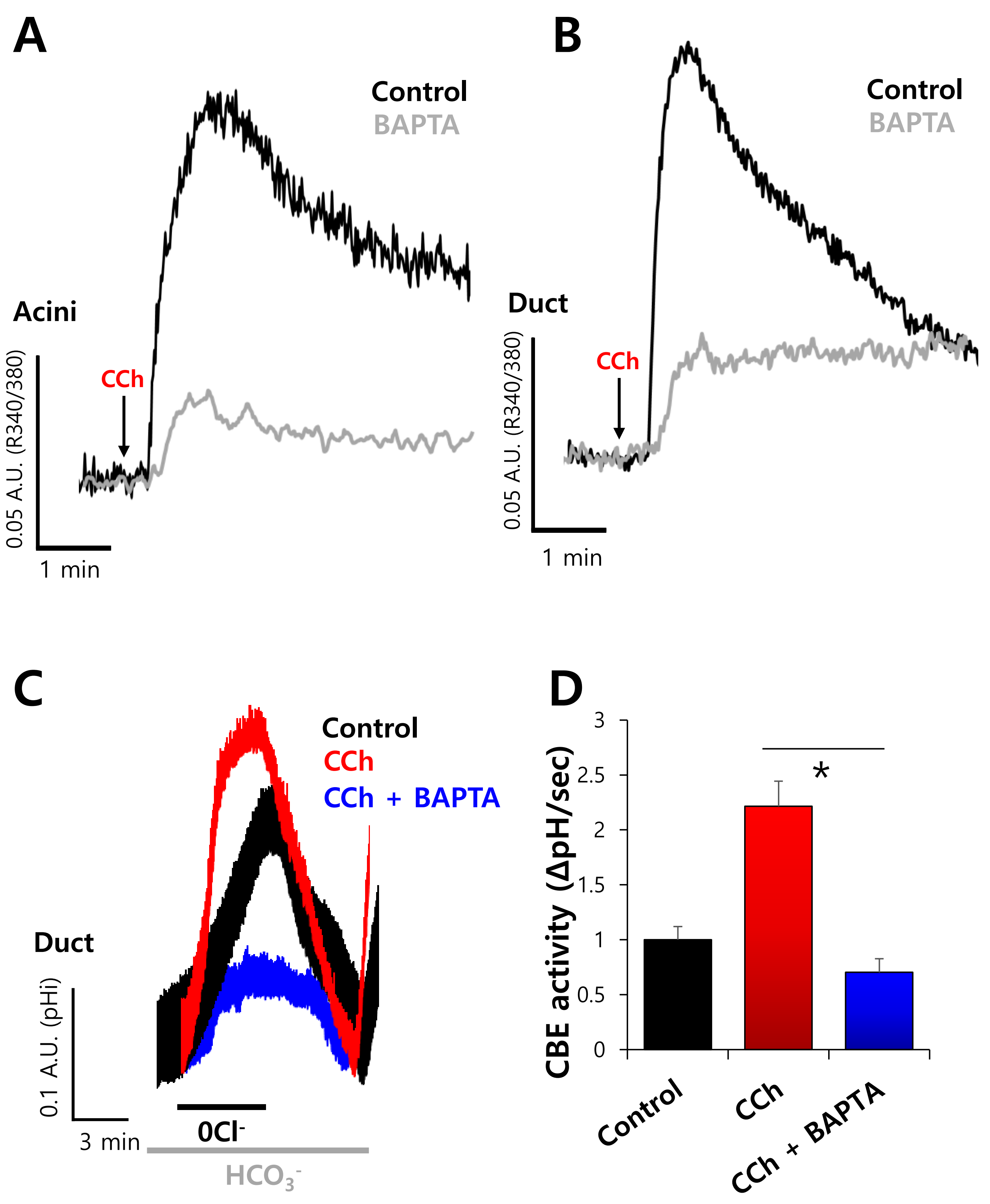

Supplement: FIGURE S1 — The pH calibration curve for (A) HEK293T, (B) A549, and primary isolated SMG (C) acini and (D) ductal cells at pH 5.5, 6.0, 6.5, 7.0, 7.5, 8.0, and 8.5. [file Data_Sheet_1.PDF]
